# From Spiradenocarcinoma to Spiradenoma: A Case of Histopathological Diagnostic Reclassification

**DOI:** 10.7759/cureus.119048

**Published:** 2026-11-03

**Authors:** Renata F Bressani, Laura C Barazzetti, Raíssa Kalsing, Vitória C Vieira

**Affiliations:** 1 Surgery, Hospital São Lucas da PUCRS, Porto Alegre, BRA; 2 General Practice, Pontifícia Universidade Católica do Rio Grande do Sul, Porto Alegre, BRA

**Keywords:** adnexal neoplasm, diagnostic reclassification, eccrine, spiradenocarcinoma, spiradenoma

## Abstract

Spiradenocarcinoma is a rare malignant cutaneous adnexal neoplasm that may arise from a preexisting spiradenoma. Distinguishing benign from malignant lesions can be challenging, and diagnostic classification has important implications for treatment and follow-up.

A 37-year-old woman presented with an approximately 3-cm erythematous-violaceous nodule on the right ankle that had been present for five years and had recently grown rapidly. The initial histopathological assessment described a malignant epithelial neoplasm compatible with spiradenocarcinoma associated with a benign spiradenoma component. The patient underwent wider surgical re-excision with clinical margins exceeding 1 cm, and computed tomography of the chest, abdomen, and pelvis showed no metastatic disease. No residual neoplasm was identified in the re-excision specimen. Subsequent review of the original histological slides at a second laboratory identified a well-circumscribed, lobulated, solid-cystic dermal lesion with two distinct epithelial cell populations and an interspersed lymphocytic infiltrate, without infiltrative growth, nuclear atypia, mitotic activity, or necrosis in the reviewed material. The diagnosis was revised to benign spiradenoma. Immunohistochemistry supported the diagnosis of an eccrine neoplasm.

This case illustrates the diagnostic challenges in distinguishing spiradenoma from spiradenocarcinoma and the clinical implications of discrepant histopathological assessments. Morphological evaluation remains central to classification, while immunohistochemistry provides complementary information.

## Introduction

Spiradenocarcinoma is an extremely rare cutaneous adnexal neoplasm that most commonly arises from the malignant transformation of a preexisting eccrine spiradenoma, although de novo development has also been reported [1-3]. Benign spiradenomas typically present as solitary, slowly growing nodules. Pain, rapid growth, ulceration, or other changes in clinical behavior warrant further investigation but are not sufficient to establish malignancy [4-6].

Histopathological assessment is essential for distinguishing spiradenoma from spiradenocarcinoma. Features supporting malignancy include infiltrative growth, cytologic atypia, increased mitotic activity, and necrosis. However, low-grade spiradenocarcinomas may lack some of these overtly malignant features, making the distinction challenging when atypia or mitotic activity is limited and the overall architecture is not unequivocally malignant [1-4,7]. Consequently, careful assessment of the tumor’s architecture and cytological features is particularly important when the initial diagnosis is uncertain. This distinction has direct clinical implications. A diagnosis of spiradenocarcinoma may prompt wider surgical excision, staging investigations to assess for metastatic disease, and closer clinical or radiological follow-up [2,8], whereas a confidently established benign spiradenoma generally does not warrant the same oncological approach.

We report the case of a 37-year-old woman whose ankle lesion was initially diagnosed as spiradenocarcinoma but was subsequently reclassified as spiradenoma following histopathological review.

## Case presentation

A 37-year-old woman with no relevant medical history presented with an approximately 3-cm, well-circumscribed, smooth, erythematous-violaceous nodule on the right ankle (Figure 1). The lesion had been present for approximately five years, with recent rapid growth. The patient reported no pain, tenderness, ulceration, or bleeding. No palpable regional lymphadenopathy was identified on clinical examination.

**Figure 1:**
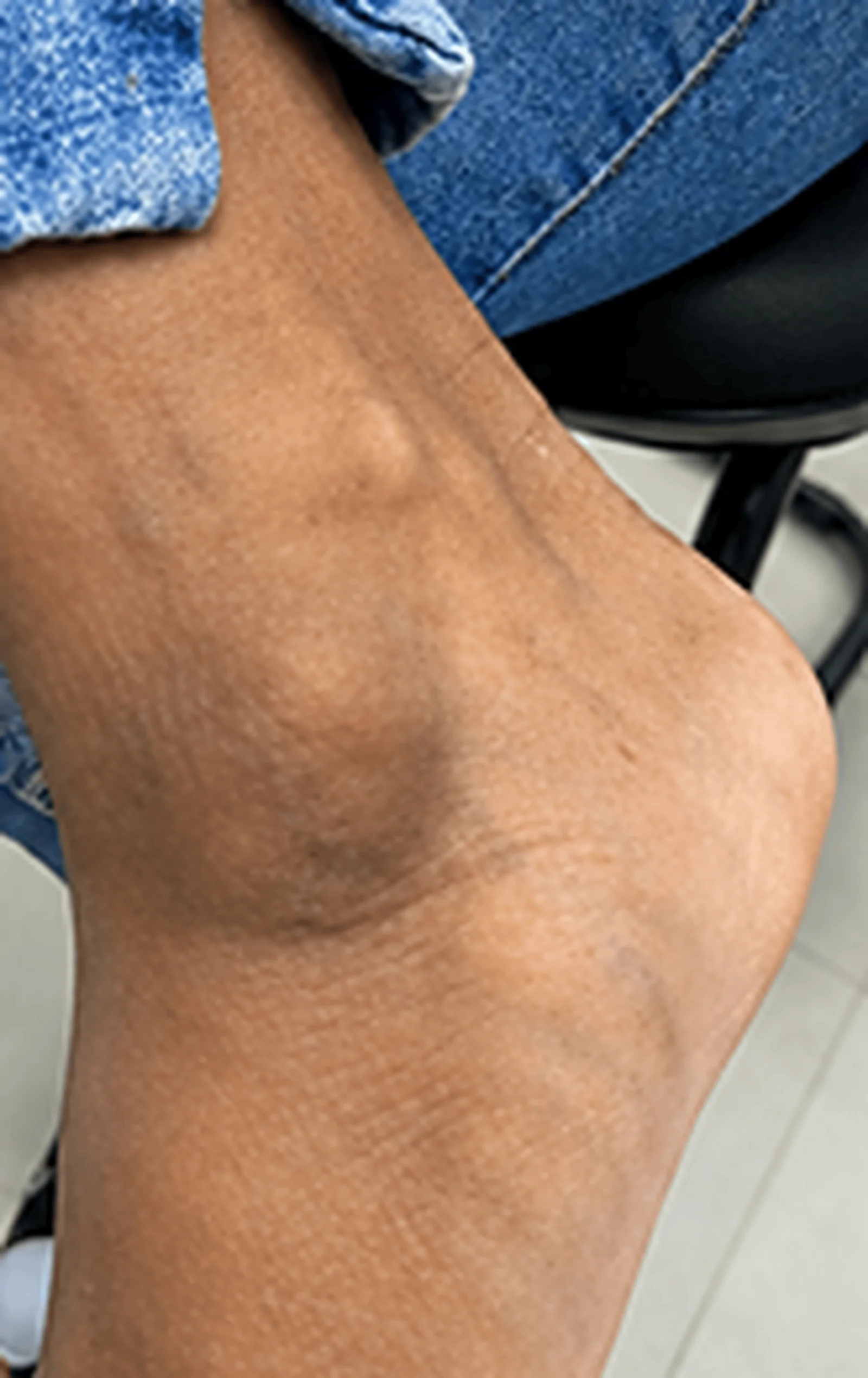
Clinical presentation of the lesion. Preoperative clinical photograph showing an approximately 3-cm, well-circumscribed, smooth, erythematous-violaceous nodule on the right ankle.

The lesion was initially excised, and histopathological examination at the original laboratory was reported as a malignant epithelial neoplasm compatible with spiradenocarcinoma, associated with a benign spiradenoma component. The available report did not specify the morphological criteria supporting the diagnosis of malignancy. Immunohistochemical analysis showed positivity for CK7 and epithelial membrane antigen (EMA) in the epithelial component and for alpha-smooth muscle actin (α-SMA) in the myoepithelial component, with negative staining for desmin and CD34. The reported Ki-67 proliferation index was 5%. According to the initial report, the benign component extended to all surgical margins, whereas the component interpreted as malignant did not involve the margins.

Given the initial diagnosis of spiradenocarcinoma, the patient underwent a wider surgical re-excision with clinical margins exceeding 1 cm, as Mohs micrographic surgery was not available. Histopathological examination of the re-excision specimen revealed no residual neoplasm, and the final surgical margins were reported as negative. Computed tomography (CT) of the chest, abdomen, and pelvis was performed for staging after the initial malignant diagnosis and showed no evidence of metastatic disease.

The original histological slides were subsequently reviewed at a second laboratory affiliated with the outpatient clinic. No additional tissue sections were obtained. On review, the lesion was characterized as a well-circumscribed, solid-cystic dermal nodule with a lobulated architecture, composed of two distinct populations of epithelial cells with interspersed lymphocytes (Figure 2). No infiltrative growth, nuclear atypia, mitotic activity, or necrosis was identified in the reviewed material. Based primarily on these morphological findings, the diagnosis was revised to benign spiradenoma.

**Figure 2:**
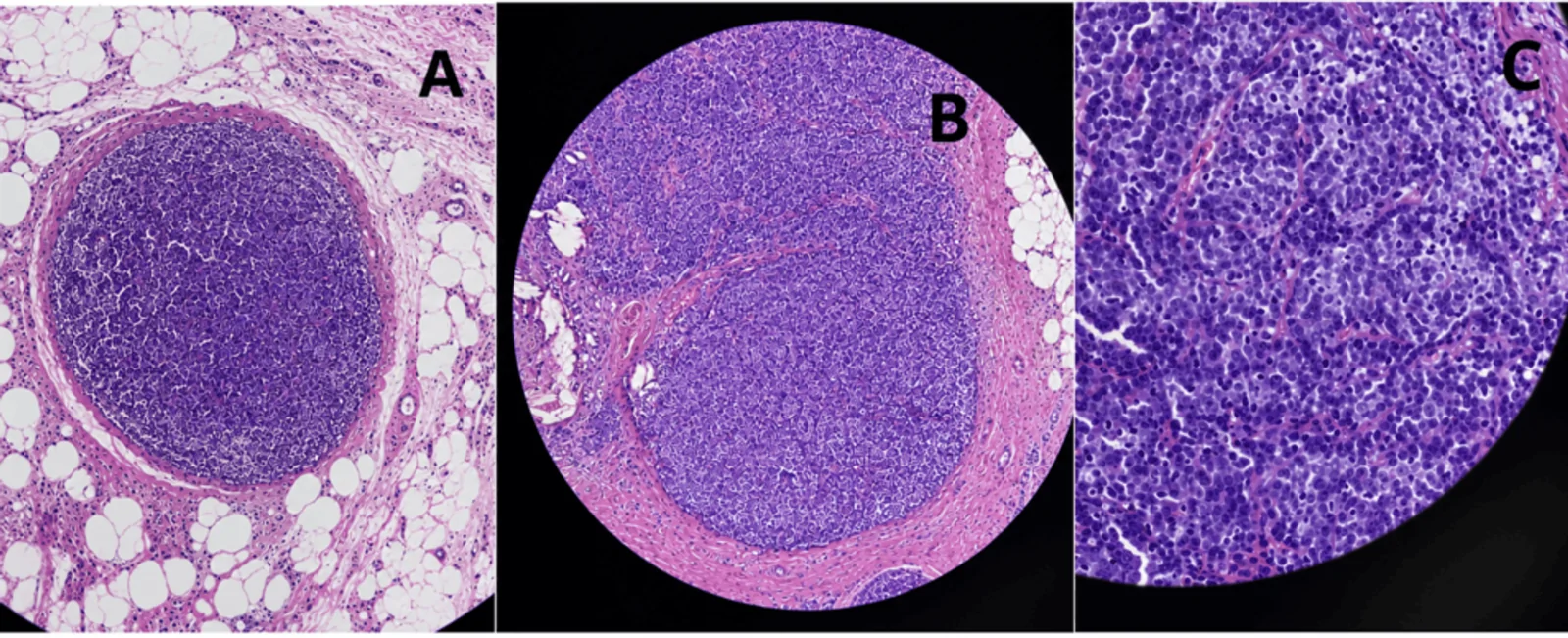
Histopathological features of the lesion on review. (A) Hematoxylin and eosin (H&E) staining, ×40, showing a well-circumscribed, lobulated dermal nodule adjacent to subcutaneous adipose tissue. (B) H&E staining, ×100, demonstrating basaloid epithelial cells arranged in nests and cords, with fibrous to hyalinized stroma and an interspersed lymphocytic infiltrate. (C) H&E staining, ×400, suggesting two distinct cellular populations, consisting of smaller cells with hyperchromatic nuclei and larger cells with paler cytoplasm, along with interspersed hyaline material. On review, the morphological findings were considered consistent with benign spiradenoma.

Immunohistochemical assessment at the second laboratory showed positivity for polyclonal S100 and SOX10 in the myoepithelial component, while CD117 and carcinoembryonic antigen (CEA) were negative (Figure 3). The reported Ki-67 proliferation index was 10%. Although the indices reported by the two laboratories differed (5% and 10%), both reports were based on the same tissue blocks. Neither report specified the Ki-67 counting method or whether the reported value represented a hotspot or an overall estimate. Accordingly, the difference in proliferation indices could not be reliably interpreted in isolation, and the diagnostic reclassification was based primarily on the morphological reassessment rather than on immunohistochemistry alone.

**Figure 3:**
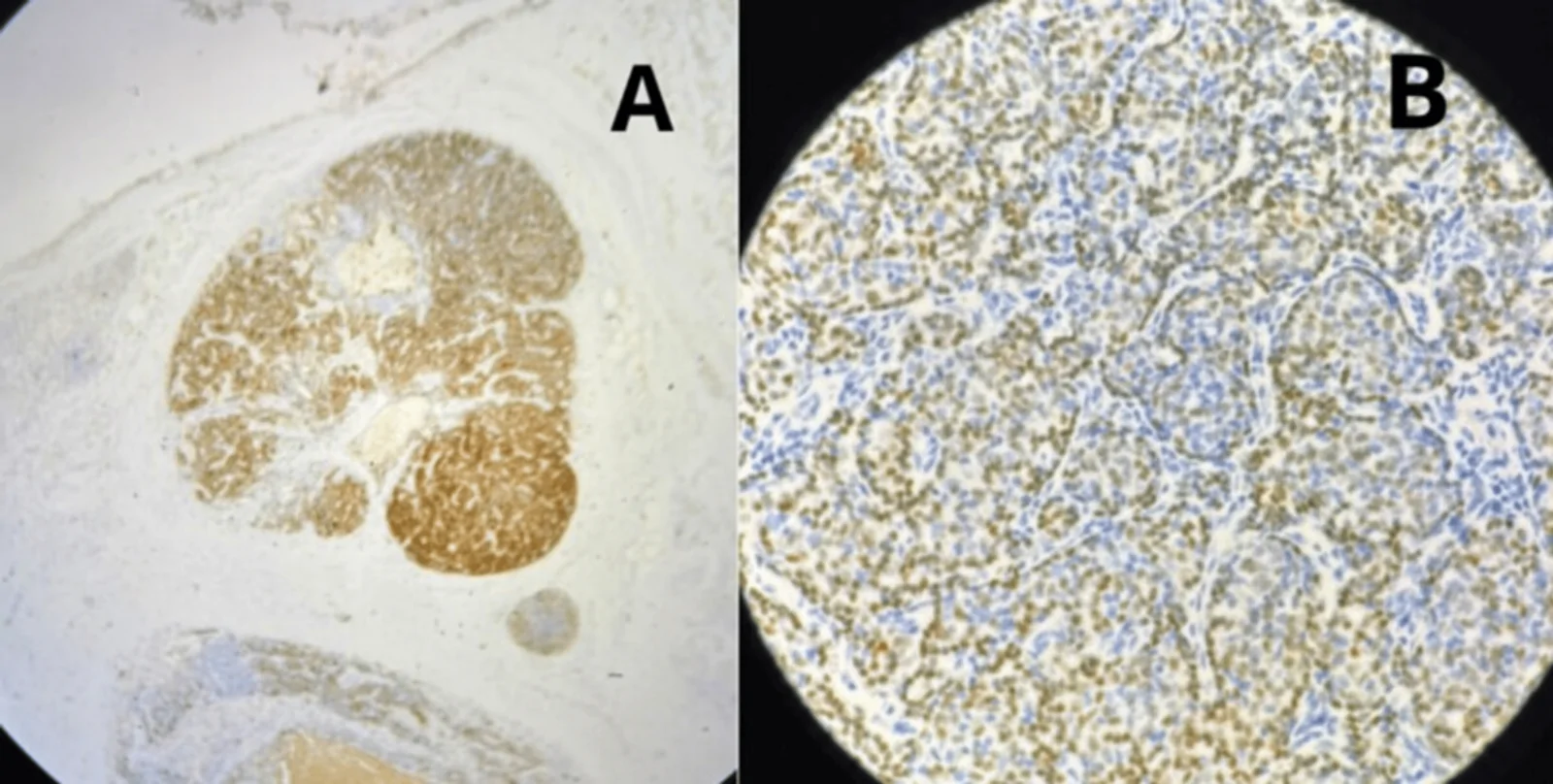
Immunohistochemical findings in the reviewed specimen. (A) Polyclonal S100 immunostaining, ×100, showing positivity in the myoepithelial component. (B) SOX10 immunostaining, ×400, demonstrating positivity in the myoepithelial component. These findings support the identification of the myoepithelial component and should be interpreted in conjunction with the histomorphological findings.

The patient remains under clinical and radiological follow-up. Repeat CT imaging approximately one year after surgery showed no evidence of recurrence. At the most recent clinical assessment, conducted three months before the current report, no clinical recurrence was identified. The interval from the initial consultation to the most recent assessment was approximately two years, including one year and seven months since surgery. Follow-up has been continued in view of the initial malignant diagnosis, the discrepancy between the pathological assessments, and the limited evidence available to guide surveillance in this specific clinical context.

## Discussion

Eccrine spiradenoma is a benign cutaneous adnexal neoplasm that typically presents as a solitary dermal nodule, whereas spiradenocarcinoma is a rare malignant neoplasm that may arise through malignant transformation of a preexisting spiradenoma or, less commonly, develop de novo [1-4]. Although clinical changes such as rapid growth may raise suspicion for malignancy, these findings are not specific, as benign spiradenomas may also undergo changes in size or presentation [4-6]. Therefore, clinical behavior should prompt appropriate investigation but cannot independently establish the diagnosis. Histopathological examination remains essential for distinguishing these entities.

The distinction between spiradenoma and spiradenocarcinoma can be particularly challenging in suspected low-grade malignancies, in which overt cytological atypia or other conventional features of malignancy may be subtle. Features that support malignant transformation include loss of the dual cell population, infiltrative growth, cytological atypia, increased mitotic activity, necrosis, and, in some cases, an abrupt transition between benign and malignant components [1-4,8]. However, these findings must be interpreted in the context of the overall tumor architecture and cytological characteristics rather than in isolation. Eccrine spiradenoma exhibits a range of histomorphological patterns that should be considered when evaluating suspected malignant transformation. Classically, it presents as a well-circumscribed, basophilic dermal nodule, although multinodular growth and extension into the subcutis may also occur. Tumor cells may be arranged in compact nests, sheets, trabeculae, or interconnected cords forming a reticular pattern. A characteristic feature is the presence of two distinct cellular populations: smaller cells with hyperchromatic nuclei and larger cells with paler cytoplasm. Although these populations may show a peripheral-to-central distribution within tumor nests, this arrangement is not necessarily uniform throughout the lesion. Intratumoral lymphocytes are another characteristic finding, while ductal structures, basement membrane-like hyaline material, and stromal changes ranging from edema to hyalinization may be variably present [5-7].

In the present case, the initial pathological report described a malignant epithelial neoplasm compatible with spiradenocarcinoma associated with a benign spiradenoma component. However, the available report did not specify the morphological criteria supporting the malignant interpretation. On subsequent review of the original slides, the lesion was characterized by a well-circumscribed, solid-cystic dermal nodule with a lobulated architecture, two distinct epithelial cell populations, and an interspersed lymphocytic infiltrate. Infiltrative growth, nuclear atypia, moderate mitotic activity, and necrosis were not identified in the reviewed material. These findings supported reclassification as benign spiradenoma.

Immunohistochemistry provided complementary information regarding the cellular components of the lesion but was not independently diagnostic. In the initial assessment, CK7 and EMA positivity supported the epithelial component, while α-SMA positivity supported myoepithelial differentiation. In the subsequent assessment, S100 and SOX10 positivity further supported the identification of the myoepithelial component. These findings should be interpreted alongside the morphological features, as immunohistochemical profiles may overlap among cutaneous adnexal neoplasms and do not independently establish benign or malignant behavior [7,8].

The Ki-67 proliferation indices reported by the two laboratories were 5% and 10%, respectively. Both assessments were based on the same tissue blocks; however, the available reports did not specify the counting methodology. Consequently, the difference cannot be reliably interpreted as evidence of increased proliferative activity or malignancy. In this case, the reclassification was based primarily on the morphological findings identified during the second review rather than on the difference in Ki-67 or the immunohistochemical results alone.

The initial diagnosis also had important implications for the clinical management in this case. Because spiradenocarcinoma may warrant wider excision and evaluation for metastatic disease [8], the patient underwent re-excision with clinical margins exceeding 1 cm, as Mohs micrographic surgery was unavailable, and CT imaging of the chest, abdomen, and pelvis for staging. The re-excision specimen showed no residual neoplasm, and the final surgical margins were negative. CT imaging showed no evidence of metastatic disease. Given the residual diagnostic uncertainty arising from the discrepancy between the initial and revised histopathological assessments, long-term clinical follow-up was deemed appropriate in this case. This decision reflects the specific circumstances of the present case and should not be interpreted as a general recommendation for long-term surveillance in patients with benign spiradenoma.

At the most recent assessment, approximately one year and seven months after surgery, no clinical recurrence had been identified, and repeat CT imaging performed approximately one year after surgery showed no evidence of recurrence. The absence of recurrence during the documented follow-up period does not establish long-term outcomes.

This case highlights the importance of correlating clinical findings with tumor architecture, cytological features, and complementary immunohistochemical results when evaluating rare cutaneous adnexal neoplasms. When a pathological diagnosis has substantial implications for treatment, review of the available histological material may help clarify diagnostic uncertainty. 

## Conclusions

This case highlights the diagnostic challenges in distinguishing spiradenoma from spiradenocarcinoma and the potential clinical implications of histopathological reclassification. Careful assessment of tumor morphology, supported by complementary immunohistochemical findings, is essential when the diagnosis has significant implications for treatment. In this case, the discrepancy between the initial and revised pathological assessments, together with the residual diagnostic uncertainty, led to the decision to maintain long-term clinical follow-up. However, the findings of a single case do not establish a general surveillance strategy for benign spiradenoma, and the absence of recurrence during the documented follow-up period does not establish long-term outcomes.
